# RGC-32 Acts as a Hub to Regulate the Transcriptomic Changes Associated With Astrocyte Development and Reactive Astrocytosis

**DOI:** 10.3389/fimmu.2021.705308

**Published:** 2021-07-29

**Authors:** Alexandru Tatomir, Austin Beltrand, Vinh Nguyen, Jean-Paul Courneya, Dallas Boodhoo, Cornelia Cudrici, Dafin F. Muresanu, Violeta Rus, Tudor C. Badea, Horea Rus

**Affiliations:** ^1^Department of Neurology, University of Maryland, School of Medicine, Baltimore, MD, United States; ^2^Department of Neurosciences, “Iuliu Hatieganu” University of Medicine and Pharmacy, Cluj-Napoca, Romania; ^3^Department of Medicine, Division of Rheumatology and Clinical Immunology, University of Maryland, School of Medicine, Baltimore, MD, United States; ^4^Health Sciences and Human Services Library, University of Maryland, Baltimore, MD, United States; ^5^Translational Vascular Medicine Branch, National Heart, Lung and Blood Institute, National Institutes of Health, Bethesda, MD, United States; ^6^Retinal Circuit Development and Genetics Unit, N-NRL, National Eye Institute, Bethesda, MD, United States; ^7^Research and Development Institute, Faculty of Medicine, Transylvania University of Brasov, Brasov, Romania; ^8^Research Service, Veterans Administration Maryland Health Care System, Baltimore, MD, United States

**Keywords:** RGC-32, astrocyte, radial glia, experimental autoimmune encephalomyelitis, RNA sequencing, TGF-β, CD133, HOPX

## Abstract

Response Gene to Complement 32 (RGC-32) is an important mediator of the TGF-β signaling pathway, and an increasing amount of evidence implicates this protein in regulating astrocyte biology. We showed recently that spinal cord astrocytes in mice lacking RGC-32 display an immature phenotype reminiscent of progenitors and radial glia, with an overall elongated morphology, increased proliferative capacity, and increased expression of progenitor markers when compared to their wild-type (WT) counterparts that make them incapable of undergoing reactive changes during the acute phase of experimental autoimmune encephalomyelitis (EAE). Here, in order to decipher the molecular networks underlying RGC-32’s ability to regulate astrocytic maturation and reactivity, we performed next-generation sequencing of RNA from WT and RGC-32 knockout (KO) neonatal mouse brain astrocytes, either unstimulated or stimulated with the pleiotropic cytokine TGF-β. Pathway enrichment analysis showed that RGC-32 is critical for the TGF-β-induced up-regulation of transcripts encoding proteins involved in brain development and tissue remodeling, such as axonal guidance molecules, transcription factors, extracellular matrix (ECM)-related proteins, and proteoglycans. Our next-generation sequencing of RNA analysis also demonstrated that a lack of RGC-32 results in a significant induction of WD repeat and FYVE domain-containing protein 1 (Wdfy1) and stanniocalcin-1 (Stc1). Immunohistochemical analysis of spinal cords isolated from normal adult mice and mice with EAE at the peak of disease showed that RGC-32 is necessary for the *in vivo* expression of ephrin receptor type A7 in reactive astrocytes, and that the lack of RGC-32 results in a higher number of homeodomain-only protein homeobox (HOPX)^+^ and CD133^+^ radial glia cells. Collectively, these findings suggest that RGC-32 plays a major role in modulating the transcriptomic changes in astrocytes that ultimately lead to molecular programs involved in astrocytic differentiation and reactive changes during neuroinflammation.

## Introduction

Astrocytes are important players in the cellular mechanisms of multiple sclerosis (MS) pathogenesis and its animal model, experimental autoimmune encephalomyelitis (EAE), both of which are chronic, demyelinating disorders of the central nervous system (CNS) (1). The ability of astrocytes to undergo complex molecular and morphological changes, collectively termed reactive astrogliosis (2–4), place them at the crossroads between inflammatory resolution and repair, on one hand, and the neurodegenerative processes that underlie MS progression on the other (5). The duality of reactive astrocytes in shaping EAE disease evolution is evidenced by numerous studies showing that they can be both beneficial and detrimental (6). The deleterious effects of reactive astrocytes include their ability to secrete pro-inflammatory cytokines and chemokines, effectively recruiting inflammatory cells into CNS, and their disruption of the blood-brain barrier (BBB), generation of reactive oxygen and nitrogen species, and secretion of factors with neurotoxic potential (7, 8). On the other hand, other studies have shown that reactive astrocytes can be beneficial as well, through their ability to form a glial scar around the inflammatory infiltrate, successfully containing the inflammation and stopping the infiltration into surrounding, healthy tissue, as well as their promotion of BBB repair and secretion of neuroprotective factors (6, 9, 10). The glial scar itself poses a particular problem, because although it is capable of providing a physical barrier against inflammatory cell invasion, such a barrier can eventually prove detrimental by inhibiting axonal regeneration and remyelination during chronic EAE (11). However, this view has recently been challenged by studies showing that axonal regeneration can occur in a progliotic environment (12, 13). Therefore, the heterogeneity of reactive astrocytosis underlies the delicate balance between protective and detrimental effects and may dictate the direction of the outcome, either toward injury resolution and repair or disease progression and permanent damage (14, 15). In any case, given the positive correlation between disease severity and astrocytic reactivity, reactive astrocytes are now regarded as major players in driving MS progression (15, 16).

We previously showed that Response Gene to Complement 32 (RGC-32) is an essential component of the signaling pathways downstream of TGF-β that are involved in the synthesis of extracellular matrix (ECM) constituents, such as fibronectin and collagens type I, IV, and V, because it physically interacts with SMAD3 and regulates its nuclear translocation (17). Recently, we have also demonstrated that a lack of RGC-32 ameliorates EAE and results in a defect in the ability of Th cells to differentiate toward the Th17 subset (18). Moreover, our results have shown that in the absence of RGC-32, spinal cord astrocytes are unable to acquire a reactive phenotype during acute EAE, remaining instead in a progenitor state reminiscent of radial glia. These radial glia-like cells from RGC-32 knockout (KO) mice are characterized by an elongated bipolar shape and an increased expression of progenitor markers such as vimentin and fatty acidic binding protein (FABP7) (19). They also exhibit an increased proliferative capacity, as shown by their increased expression of the cell proliferation marker Ki-67 when compared to wild-type (WT) mice (19). *In vitro* studies have shown that RGC-32 regulates the synthesis and secretion of growth factors such as connective tissue growth factor (CTGF), insulin-like growth factor 1 (IGF-1), and IGF binding proteins (IGFBPs) (19). Our experiments have also demonstrated that RGC-32 regulates the nuclear translocation of signal transducer and activator of transcription 3 (STAT3), a transcription factor critical for astrogliogenesis and reactive astrocytosis (19). Based on these observations, we have concluded that RGC-32 is necessary for astrocytes to reach maturity and to undergo reactive changes at the peak of EAE, and a lack of RGC-32 confers a protective phenotype against EAE in part by suppressing the development of a pathogenic astrocytic phenotype.

However, we still lack a clear picture of the overall molecular programs underlying RGC-32’s contribution to astrogliogenesis and reactive astrocytosis. We have now performed next-generation sequencing of RNA from neonatal WT and RGC-32 KO astrocytes and show that RGC-32 deletion has a profound effect on the ability of TGF-β to induce transcriptomic changes in astrocytes. We found that in RGC-32 KO astrocytes, the transcriptomic programs associated with brain development and neurogenesis are significantly affected, and the transcription of genes encoding axonal guidance molecules (AGM) are particularly impaired, as are genes involved in astrocyte development. Moreover, the lack of RGC-32 leads to a major up-regulation in the expression of two genes commonly expressed in neural stem cells and astrocyte progenitors. We show that the lack of RGC-32 also results in higher numbers of cells in two subpopulations of spinal cord radial glia-like cells at the peak of EAE. Taken together, these results suggest that RGC-32 is an important gene that orchestrates the transcriptomic changes undergone by astrocytes as they mature and adopt a reactive phenotype during EAE.

## Materials and Methods

### Mice

All mice used in this study were on a C57BL/6 background and were 6–12 weeks of age. The mice were housed under pathogen-free conditions. RGC-32 KO mice were generated as described previously (20), and WT C57BL/6 littermates were used as controls. All procedures were approved by the University of Maryland School of Medicine Office of Animal Welfare Assurance.

### Induction and Evaluation of EAE

WT and RGC-32 KO female mice (8–10 weeks old) were injected subcutaneously in two locations in the dorsal flank with an emulsion containing 200 μg of MOG_35–55_ (Anaspec, Fremont, CA) and complete Freund’s adjuvant (CFA) (Difco, Detroit, MI) as previously described (18). Pertussis toxin (400 ng; List Biological Laboratories, Campbell, CA) was administered intraperitoneally on days 0 and 2. Mice were monitored daily, and the disease was scored on a scale of 0–5 as follows: 1 - limp tail; 2 - hindlimb paresis; 3 - hindlimb paralysis; 4 - tetraplegia; 5 - moribund. Age- and sex-matched uninjected mice were used as controls (day 0).

### Primary Astrocyte Isolation

Neonatal astrocytes were purified from the brains of 1-day-old WT and RGC-32 KO mouse pups as previously described (17). After removal of the meninges, the brains were minced and sequentially passed through nylon meshes. The resulting cell suspensions were plated onto 75-cm^2^ plates in DMEM/Ham’s F-12 medium containing 10% fetal bovine serum (FBS) for 2 weeks. Oligodendrocyte precursor cells and neurons were separated from the astrocyte monolayer by shaking overnight at 200 rpm on a rotary shaker. The non-adherent cell suspension was discarded, and adherent astrocytes were then maintained in DMEM/Ham’s F-12 medium containing 10% FBS. More than 97% of the cells isolated expressed the astrocyte marker glial fibrillary acidic protein (GFAP). Astrocytes were serum-starved overnight prior to stimulation with 10 ng/ml TGF-β (Gemini Bio, West Sacramento, CA) for 24 h.

### RNA Sequencing

RNASeq data were derived from WT and RGC-32 KO astrocytes, either untreated or treated with TGF-β. For each genotype and treatment, two biological replicates were analyzed, and a total amount of 1 µg of RNA was used for each sample. RNA quality was determined by Genewiz NGS Laboratory (South Plainfield, NJ), and RNA integrity numbers were between 8.5 and 9.6. Unstranded sequencing resulted in about 45 million reads/sample, with 33 million unique reads for each. Alignments were performed using the VIPER pipeline (implementing the STAR aligner) (21, 22) on the NIH High Performance Computing Biowulf cluster (http://hpc.nih.gov). Most of the reads aligned to the open reading frame, with fewer than 5% intronic reads, about 20% 3’UTR alignments, and about 2.5% 5’UTR hits. rRNA represented about 1.5% across all samples. Differential gene expression was performed using both limma and DESEQ2. Further analysis in this paper represents differentially expressed genes (DEG) with at least a 1.5x differential -fold change-either up-regulated or down-regulated relative to unstimulated (corresponding to |log_2_ -fold change| ≥ 0.6) and a false discovery rate (FDR) < 0.05, after Bonferroni correction using the DESEQ2 protocol (23). Gene expression data are available on the Gene Expression Omnibus (GEO, http://www.ncbi.nlm.nih.gov/geo; accession number GSE173782).

### Functional Enrichment Analysis

Pathway enrichment was performed by using the g:Profiler web server (https://biit.cs.ut.ee/gprofiler/gost). The lists of genes were uploaded into the g:GOSt platform for functional profiling. For the statistical significance threshold, we used Bonferroni correction with an FDR of 0.05. We then generated Gene Ontology (GO) categories for Biological Process (BP), Molecular Function (MF), and Cellular Component (CC), as well as Reactome biological pathways, according to website’s data retrieved from curated databases (24).

### Generation of a Global Pathway Enrichment Map

The list of the BP genes generated as described above was uploaded into the EnrichmentMap plugin of the Cytoscape platform (25). The enrichment map was generated using a gene-set filter based on an FDR cutoff of 0.05 for the number of nodes and a gene-set similarity filter based on the Jaccard coefficient of 0.25 for the number of edges. Each node represents an individual BP, and each edge between nodes represents gene overlap. We then arranged the network according to the yFiles Radial Layout algorithm and manually clustered the functionally and annotation-similar nodes.

### Connectivity Analysis of Gene Networks

Genes of interest were entered into the STRING online platform using the “Multiple proteins” input (https://string-db.org). A full network of interconnected nodes was then generated based on both functional and physical gene product associations, according to STRING’s algorithms computed from curated experimental data (26). The type of interactions between the query genes was set to confidence, with line thickness representing the strength of data support, based on a minimum interaction score of 0.5 and an FDR stringency of 0.05, according to STRING website’s classification. The networks were then loaded into Cytoscape to allow for a better visualization of the interactions.

### RNA Isolation and cDNA Synthesis

Total RNA from mouse astrocytes was purified using an RNeasy Mini Kit (Qiagen, Germantown, MD) according to the manufacturer’s instructions: 0.5 μg of RNA per sample was reverse-transcribed in a mixture containing reverse transcriptase buffer (Promega, Madison, WI), dNTPs (Promega), and random primers (Invitrogen, Carlsbad, CA). The RNA was denatured by incubation at 65°C for 5 min. M-MLV reverse transcriptase (Promega) was then added, and the reaction mixture was incubated at 37°C for 1 h. The reaction was terminated by incubation of the mixture at 95°C for 5 min.

### Quantitative Real-Time PCR

Forward and reverse primers for Egfl6, Epha7, Fbln2, Fbn1, Fbn2, Hspg2, Itgb1, Plxna1, Runx2, Slit2, Spock3, Stc1, Vcan, and Wdfy1 were provided by Integrated DNA Technologies (Coralville, IA) (**Supplementary Table 1**). 18S was used as an internal control. Real-Time PCR was performed according to the manufacturer’s protocol using FastStart Universal SYBR Green Master Mix (Roche, Indianapolis, IN) and a StepOnePlus Real-Time PCR System (Applied Biosystems, Carlsbad, CA). For quantification, we used the ΔΔCT method of relative quantification as previously described (27).

### Immunohistochemistry

Control mice (day 0) and mice with EAE (day 14) were sacrificed under terminal anesthesia and perfused transcardially with 4% paraformaldehyde in PBS. Cervical spinal cords were harvested, fixed in formaldehyde, and then embedded in paraffin. Paraffin sections (5 μm) were deparaffinized and rehydrated by serial washes in xylene and alcohol (100%, 95%, 70%) and then washed in PBS. Antigen retrieval was achieved by immersion of slides in sodium citrate buffer (pH 6) and boiling at 95°C for 30 min, followed by cooling of the sections at room temperature (RT) for 20 min. The endogenous peroxidase was quenched with 3% hydrogen peroxide solution (Aqua Solutions, Deer Park, TX) for 10 min, and the non-specific binding was blocked with 10% bovine serum albumin (Gemini Bio) in PBS for 30 min. The sections were then incubated overnight at 4°C with primary antibody: polyclonal rabbit anti-CD133 IgG (Proteintech, Rosemont, IL); polyclonal rabbit anti-HOPX IgG (Proteintech); polyclonal rabbit anti-STC1 IgG (Bioss Antibodies Inc, Woburn, MA); or polyclonal rabbit anti-WDFY1 IgG (Bioss Antibodies Inc). The next day, sections were incubated with a horseradish peroxidase (HRP)-conjugated anti-rabbit IgG polymer reagent (Vector Laboratories, Burlingame, CA) for 30 min at RT. The colorimetric reactions were developed using a NovaRed Peroxidase Substrate kit (Vector Labs). The sections were subsequently washed in distilled water and counterstained with Harris hematoxylin (Sigma-Aldrich, St. Louis, MO), then dehydrated by serial washes in alcohol and xylene and mounted with a permanent mounting medium (Vector Labs). Controls for the specificity of each immunohistochemical reaction were performed by replacing the primary antibody with PBS or rabbit IgG. Immunostaining was independently evaluated by two investigators.

For double-staining immunohistochemistry, deparaffinization, rehydration, antigen retrieval, endogenous peroxidase quenching, and blocking were performed as described above. Sections were incubated overnight at 4°C with rabbit polyclonal anti-EPHA7 IgG (Bioss Antibodies Inc). The next day, sections were incubated with unconjugated Fab fragments of goat anti-rabbit IgG (Jackson ImmunoResearch Laboratories, West Grove, PA) for 1 h at RT for species conversion and then incubated with HRP-conjugated anti-goat IgG (Vector Labs) for 30 min at RT. The colorimetric reaction was developed using NovaRed (Vector Labs). After color development, endogenous alkaline phosphatase (AP) was inhibited with Bloxall Blocking Solution (Vector Labs) for 10 min, and the non-specific binding was blocked with bovine serum albumin 10% for 1 h at RT. The slides were then incubated with rabbit monoclonal anti-GFAP (Cell Signaling Technology, Danvers, MA) overnight at 4°C. The next day, sections were incubated with AP-conjugated goat anti-rabbit IgG (Vector Labs) for 1 h at RT, and the colorimetric reaction was developed with a Vector Blue AP Substrate Kit (Vector Labs). Slides were washed in distilled water and then mounted with an aqueous mounting medium (Vector Labs).

### Quantification of Immunohistochemistry

For WDFY1, STC1, and CD133 immunostaining, quantification was performed by manually counting the number of WDFY1^+^, STC1^+^, and CD133^+^ cells with radial morphology. For HOPX immunostaining, quantification was performed by manually counting the cells with HOPX^+^ nuclei. For EPHA7/GFAP double immunostaining, we counted the total number of EPHA7/GFAP double-positive cells. In each case, the respective cells were counted from regions of interest corresponding to white matter areas of 20x-magnified spinal cord sections. Quantification was performed in blinded fashion by using the Point Tool analysis of the ImageJ (19, 28).

### Statistical Analysis

Comparisons between multiple groups were performed using the Kruskall-Wallis test with Dunn’s multiple comparisons test. Comparisons between two groups were performed using the Mann-Whitney test. p-values < 0.05 were considered significant. Statistical analysis was performed using GraphPad Prism software, version 7.

## Results

### TGF-β Profoundly Changes the Transcriptomic Landscape of Murine Astrocytes

**Figure 1:**
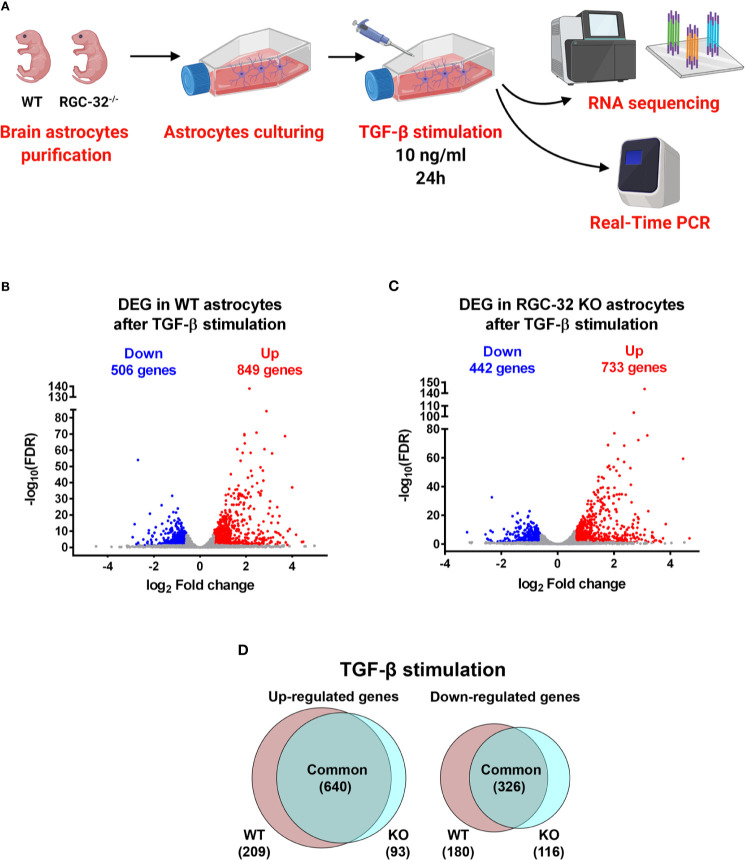
DEG in WT and RGC-32 KO astrocytes after TGF-β stimulation. **(A)** Experimental design for the RNAseq study. **(B**, **C)** Volcano plots showing the total number of DEG in WT **(B)** and RGC-32 KO astrocytes **(C)**. **(D)** Venn diagrams of the intersected DEGs showing the DEG common to both genotypes and those differentially expressed only in one genotype but not the other.

In an effort to better characterize the mechanistic pathways involved in astrocytic differentiation and reactive astrocytosis that might be differentially regulated by RGC-32, we performed comparative RNA sequencing in neonatal astrocytes purified from 1-day-old WT and RGC-32 KO mouse pups and stimulated with TGF-β (**Figure 1A**).

TGF-β stimulation resulted in a profound change in the transcriptomes of both WT and RGC-32 KO astrocytes. In WT astrocytes, our analysis revealed a total of 1355 DEG, of which 849 were up-regulated and 506 were down-regulated (**Figure 1B**). In contrast, in RGC-32 KO astrocytes, we detected 1175 DEG, of which 733 were up-regulated and 442 were down-regulated (**Figure 1C**).

### RGC-32 Deletion Has a Significant Impact on TGF-β-Induced Transcriptomic Changes in Murine Astrocytes

In order to detect the impact of RGC-32 deletion on the TGF-β-induced transcriptomic changes, we compared the identities of the DEG from WT astrocytes to those from RGC-32 KO astrocytes to reveal the DEG specific to each genotype. We found that 640 genes were up-regulated in both groups; 209 genes were up-regulated only in WT astrocytes, and 93 genes were up-regulated only in RGC-32 KO astrocytes (**Figure 1D**).

We turned our attention to the genes that were significantly up-regulated only in WT astrocytes and generated a full list of the 209 genes (**Supplementary File 1**). The first two genes in this category were fibroblast growth factor 21 (Fgf21), which encodes a growth factor with neuroprotective effects (29), and interleukin 11 (Il-11), which encodes a member of the IL-6 family of cytokines with a potential role in driving astrocyte differentiation through the activation of STAT3 (30). RGC-32 itself was the 14^th^ most up-regulated gene (Rgcc), a result that was not surprising because TGF-β is a potent inductor of its expression (17). Other potentially relevant genes included those encoding collagen isoforms (e.g., Col5a3, Col4a4), a finding that is in line with our previous observations (17).

On the other hand, among the top of those 93 genes significantly up-regulated only in RGC-32 KO astrocytes was thrombospondin 4 (Thbs4), which encodes an ECM-binding glycoprotein involved in astrogliogenesis (31) (**Supplementary File 2**). Other relevant genes in this list were doublesex and mab-3-related transcription factor-like family A1 (Dmrt1), encoding a transcription factor expressed in early progenitors during cortical neurogenesis (32); and Lamc3, encoding the gamma-3 chain of laminin, a basement membrane component involved in astrocyte migration (33).

Of the down-regulated genes, 326 genes were common to the WT and RGC-32 KO astrocytes, 180 genes were significantly down-regulated only in WT astrocytes, and 116 genes were found only in RGC-32 KO astrocytes (**Figure 1D**). Among the top genes significantly down-regulated only in WT astrocytes, we noticed epidermal growth factor-like domain-containing protein 6 (Egfl6), a member of the epidermal growth factor superfamily (**Supplementary File 3**). Intriguingly, this gene was also the most significant DEG, whose basal level was significantly higher in WT astrocytes than in RGC-32 KO astrocytes (**Supplementary File 8**).

Among the top of those 116 genes that were significantly down-regulated only in RGC-32 KO astrocytes were members of the mitogen activated protein kinase family (e.g., Map3k21), synapse components (e.g., synaptotagmin VII – Syt7) and pro-inflammatory cytokines such as Il17d, a member of the IL-17 family found to be up-regulated in peri-plaques from spinal cords of patients with progressive MS (34) (**Supplementary File 4**).

### Pathway Enrichment Analysis Reveals a Profound Effect Exerted by RGC-32 on Gene Networks Associated With Brain Development

**Figure 2:**
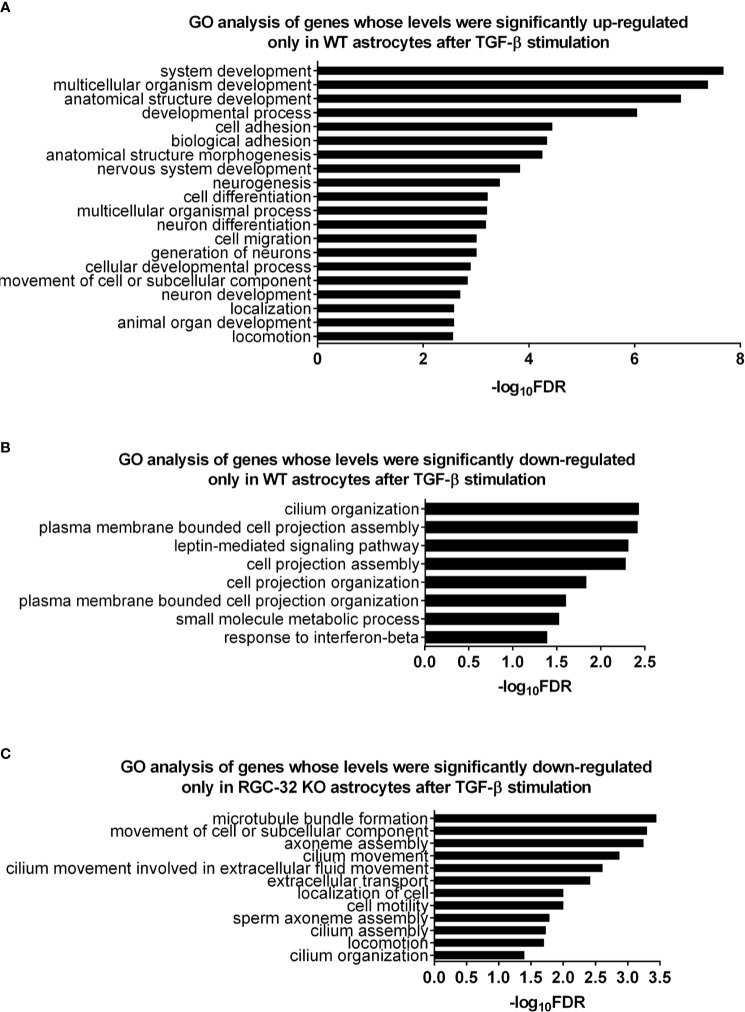
Pathway enrichment analysis for the genes differentially regulated only in one genotype after TGF-β stimulation. **(A)** Top 20 BP generated from the 209 genes up-regulated only in WT astrocytes. **(B)** All BP generated from the 180 genes down-regulated only in WT astrocytes. **(C)** All BP generated from the 118 genes down-regulated only in RGC-32 KO astrocytes. Statistical parameters: Bonferroni correction; FDR < 0.05.

In order to clarify the functional significance of the DEG detected so far and to have a more comprehensive view of the molecular events associated with these changes, we performed pathway enrichment analysis. First, we analyzed the 209 genes that were significantly up-regulated only in WT astrocytes. Our analysis revealed a total of 42 differentially regulated biological processes (BP) by using the statistical criteria depicted in *Materials and Methods*. The top 20 most differentially regulated BP were functionally related to development, morphogenesis, neurogenesis, adhesion, and cell migration (**Figure 2A**). Reactome pathway analysis detected two categories, “nervous system development” and “axonal guidance”. In terms of cellular components, the genes belonged to such categories as “cell junctions”, “adhesion”, “cell projection”, and “collagen-containing ECM”. Interestingly, some of these genes also belonged to neural compartments, such as “neural cell body” and “somatodendritic compartment”. Surprisingly, we did not detect any differentially expressed BP when we analyzed the 93 genes up-regulated only in RGC-32 KO astrocytes (see **Supplementary Data**).

We then performed pathway enrichment analysis for the 180 genes down-regulated only in WT astrocytes. Those genes belonged to eight BP related to cell projection organization (e.g., “plasma membrane bounded cell projection organization”), cilium organization, signaling (e.g., “leptin-mediated signaling pathway”, “response to interferon beta”), and metabolism (e.g., “small molecule metabolic process”) (**Figure 2B** and **Supplementary Data**). On the other hand, the genes down-regulated only in RGC-32 KO astrocytes were grouped in twelve BP that were mainly associated with cell motility (e.g., “movement of cell or subcellular component”, “locomotion”), and ciliary movement assembly and organization (e.g., “cilium movement”, “cilium assembly”, “axoneme assembly”) (**Figure 2C** and **Supplementary Data**). Cilium-associated pathways contain genes which are involved in cell migration and cytoskeletal reorganization. Due to redundancy between gene sets and GO annotations, many genes which are categorized under “GO categories” such as “cilium organization” also belong to GO categories such as “cell motility”.

**Figure 3:**
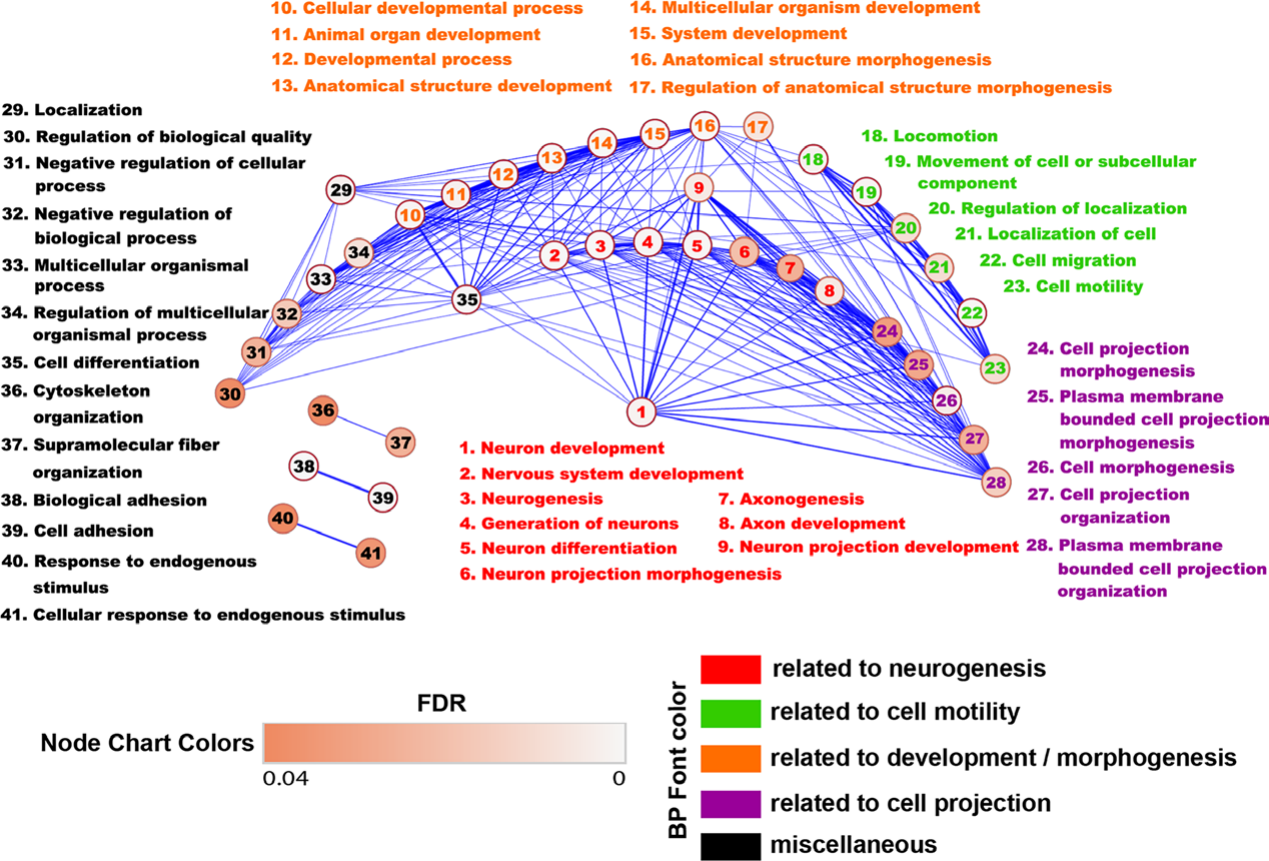
Enrichment map of the BP differentially regulated in WT astrocytes. The BP generated from genes up-regulated only in WT astrocytes were uploaded into the EnrichmentMap application of the Cytoscape software in order to generate a network in which similar BP are placed together in close association. Annotation- and functionally similar BP were clustered together into several categories. Each node represents an individual BP. The edges between nodes represent gene overlap. Edge thickness is directly proportional to the number of overlapping genes. The nodes with no connectivity were excluded. Based on their similarity, we observed that most of the BP could be clustered together into several categories, the largest ones being development and neurogenesis.

We then created an Enrichment Map from all the differentially regulated BP in WT astrocytes in order to generate a network in which similar processes are placed together in close association. Based on their similarity, we observed that most of the BP could be clustered together into several categories, the largest ones being development/morphogenesis, neurogenesis, cell motility, and cell projection (**Figure 3**). We further observed that roughly a quarter of processes were related to brain development and included terms such as” nervous system development”, “neurogenesis”, “generation of neurons” and “axonogenesis”. Other highly relevant categories were related to cell motility and cell projection and included BP such as “cell projection morphogenesis”, “movement of cell or subcellular component” and “cell migration”. Functionally similar were processes involved in cytoskeletal organization and adhesion (**Figure 3**).

Taken together, these results strongly suggest that RGC-32 is critical for TGF-β’s ability to induce the expression of gene networks that compose the cellular and molecular pathways functionally associated with brain development. On a similar note, our analysis also reveals that RGC-32 is highly relevant to cell motility and may be an important regulator of astrocyte migration, as we already demonstrated is the case for other cell types such as vascular smooth muscle cells (35) and endothelial cells (36).

### The Genes Induced Only in WT Astrocytes Include Many Genes Encoding Axonal Guidance Molecules

**Figure 4:**
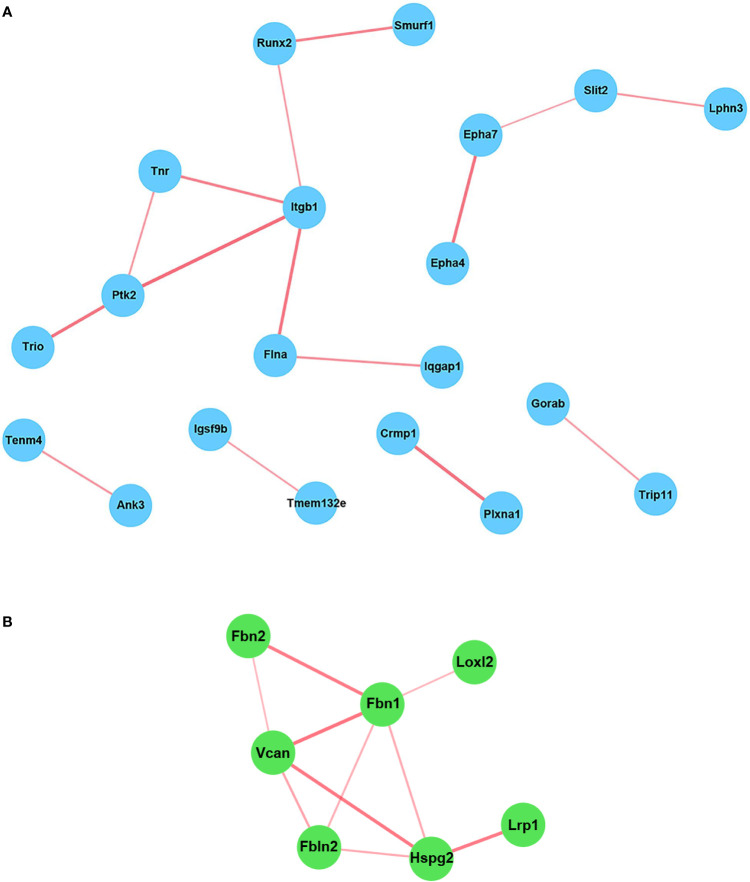
STRING network analysis of the individual genes. The genes belonging to the BP “Nervous system development” **(A)** and those whose TGF-β-regulated levels were significantly higher in WT astrocytes than in RGC-32 KO astrocytes **(B)** were uploaded into the STRING online platform in order to generate a network of functionally interconnected genes. Each node represents an individual gene. The edges between nodes represent physical and functional gene product associations. The thickness of the lines is directly proportional to the strength of the data support. The genes that showed no connectivity were excluded. Some of the functionally interacting genes encode members belonging to several families of proteins, including ephrin type A receptors (Epha4 and Epha7), beta integrins (Itgb1), Runt-related transcription factors (Runx2), plexins (Plxna1), protein tyrosine kinases (Ptk2), tenascins (Tnr), teneurins (Tenm4), and Robo-Slit signaling (Slit2) **(A)**.

**Figure 5:**
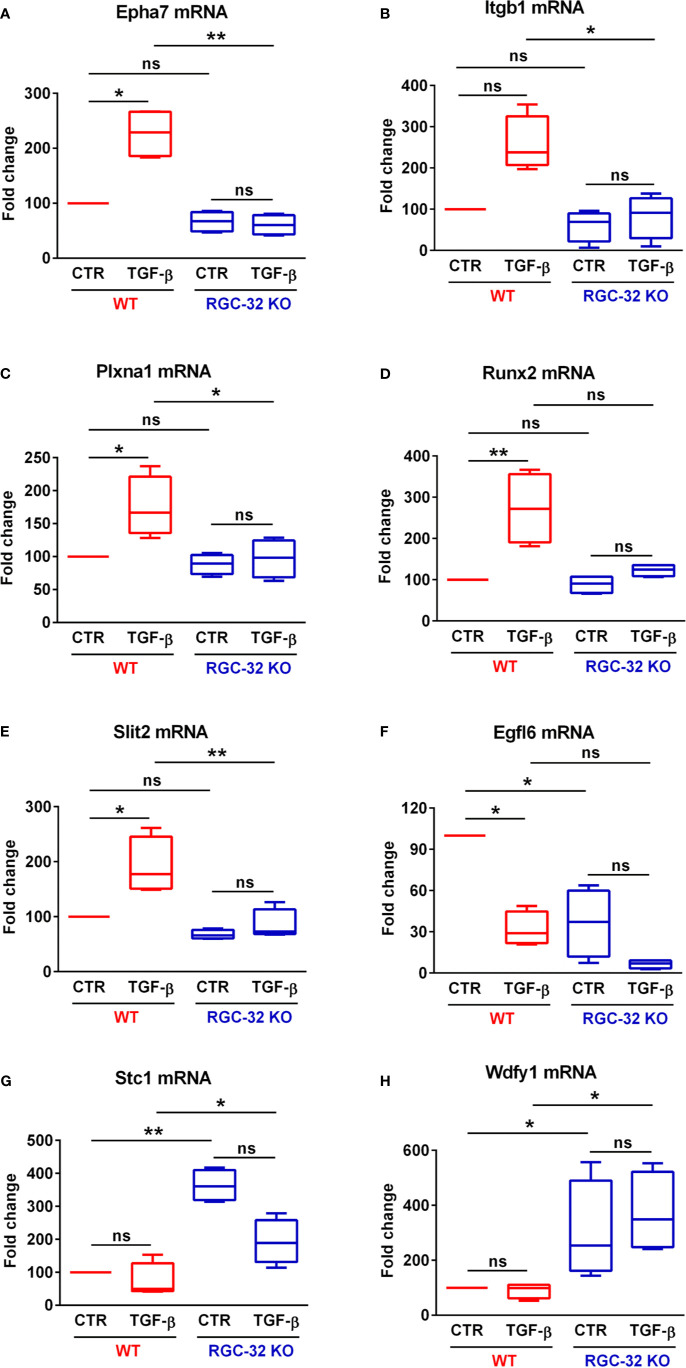
RGC-32 regulates the expression of genes associated with brain development. WT and RGC-32 KO mouse astrocytes were treated with 10 ng/ml TGF-β for 24 h and then analyzed by Real-Time PCR. Epha7 **(A)**, Plxna1 **(C)**, Runx2 **(D)**, and Slit2 **(E)** were significantly up-regulated in WT astrocytes but not in RGC-32 KO astrocytes. There was also a clear trend toward upregulation of Itgb1 levels in WT astrocytes after stimulation, although this result did not reach statistical significance **(B)**. Nevertheless, Itgb1’s induced levels were significantly higher in WT astrocytes than in RGC-32 KO astrocytes **(B)**. Egfl6 was significantly higher in unstimulated WT astrocytes when compared to their RGC-32 KO counterparts, and its expression was significantly down-regulated in WT astrocytes after TGF-β stimulation **(F)**. On the other hand, Stc1 **(G)** and Wdfy1 **(H)** levels were significantly higher in RGC-32 KO astrocytes under both basal and stimulated conditions. The expression of the mRNA in unstimulated (CTR) WT astrocytes was considered to be 100, and the results are shown as -fold change. Data are expressed as mean ± SEM (N = 4). *p < 0.05; **p < 0.01; ns, not statistically significant (Kruskall-Wallis test with Dunn’s multiple comparisons test).

Since many BP related to neurogenesis and brain development were detected as being differentially expressed only in WT astrocytes, we turned our attention to analyzing in greater detail which genes related to neural development might be of particular interest and therefore good candidates for further analysis. We chose the “nervous system development” BP and uploaded its 41 genes into the STRING online platform to generate a functional gene interaction network (**Figure 4A**). Our analysis revealed that many of the functionally interacting genes encode members of several families of guidance and patterning molecules, including ephrin type A receptors (Epha4 and Epha7), beta integrins (Itgb1), plexins (Plxna1), tenascins (Tnr), teneurins (Tenm4), and Robo-Slit signaling (Slit2). Other genes from this list encode Runt-related transcription factors (Runx2) or protein tyrosine kinases (Ptk2). Subsequent qPCR analysis confirmed the differential expression of genes such as Epha7, Plxna1, Runx2, and Slit2, with their levels being significantly up-regulated only in WT astrocytes (**Figures 5A, C–E**). We have also noticed a clear trend toward upregulation for Itgb1 in WT astrocytes, although this result did not reach statistical significance. However, when we compared the stimulated levels of Itgb1 between WT and RGC-32 KO astrocytes, we found a significantly higher level in WT astrocytes (**Figure 5B**). We have also observed that in quiescent astrocytes, RGC-32 facilitates Egfl6 expression independent of TGF-β, whereas after stimulation, RGC-32 is necessary for its TGF-β-induced down-regulation (**Figure 5F**).

### The Lack of RGC-32 Impairs the Ability of Reactive Astrocytes to Up-Regulate EPHA7 in EAE Mice

**Figure 6:**
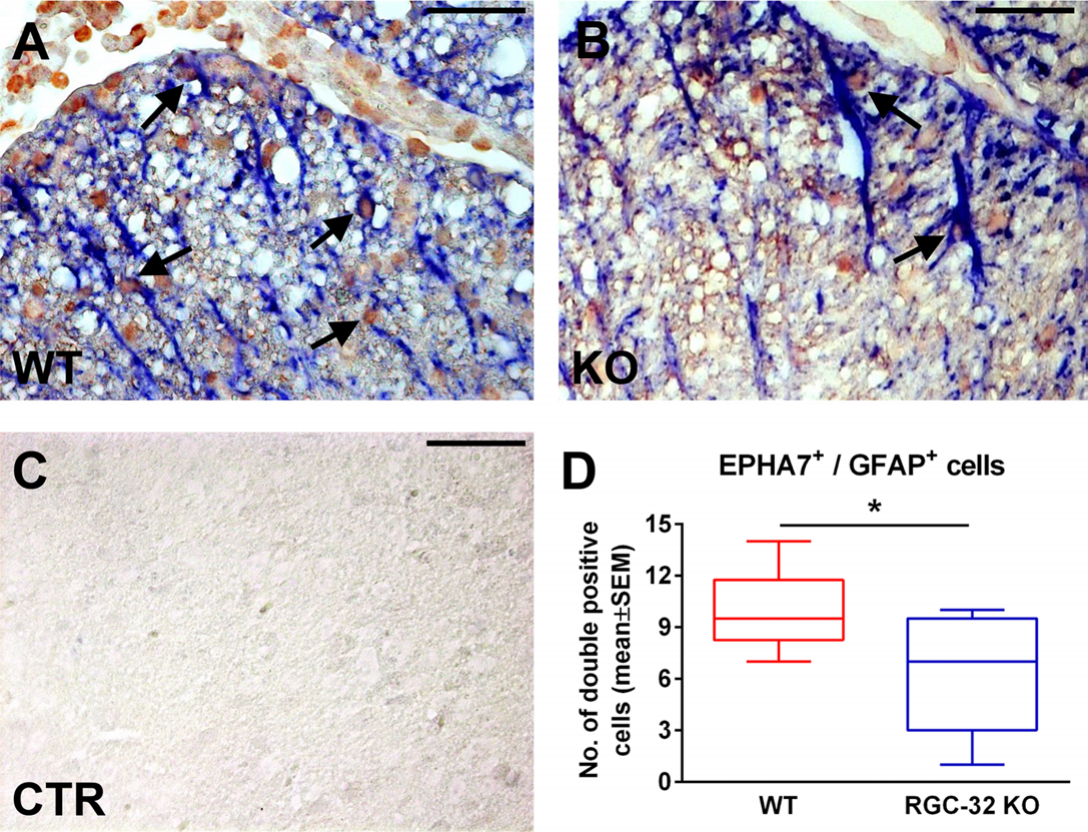
RGC-32 is necessary for the expression of EPHA7 in reactive astrocytes at the peak of EAE. Cervical spinal cords were harvested from WT and RGC-32 KO mice with EAE on day 14 and double-stained with anti-EPHA7 (red) and anti-GFAP (blue) antibody. We observed abundant EPHA7 co-localization with astrocytes in WT mice (**A**, arrows), whereas in RGC-32 KO mice, EPHA7/GFAP double-positive cells were rare (**B**, arrows). Controls (CTR) for the immunoperoxidase and AP reactions were negative **(C)**. Original magnification: x40. Scale bars: 20 µm. When we counted the total number of EPHA7/GFAP-double positive cells per microscopic field, we noticed that the number in WT mice was statistically significantly higher than in RGC-32 KO mice **(D)**. Results in **(D)** are expressed as mean ± SEM. N = 4 mice (8 microscopic areas) in WT; n = 4 mice (9 microscopic areas) in RGC-32 KO. *p < 0.05 (Mann-Whitney test).

Ephrin receptor type 7A (EPHA7) is up-regulated in reactive astrocytes and this receptor is among the ephrin receptors with the highest expression in active MS lesions (37). Based on our RNAseq results, we decided to further characterize its expression during EAE. We first induced EAE in WT and RGC-32 KO mice. As in our previous observations (18, 19), RGC-32 KO mice developed a less severe clinical picture throughout the course of disease (**Supplementary Figure 1**). We then performed double-staining immunohistochemistry for EPHA7 and GFAP on spinal cords collected at the peak of EAE (day 14). Our analysis (**Figures 6A–D**) showed an abundance of astrocytes positive for EPHA7 around the inflammatory infiltrate in the WT mice (**Figure 6A**, arrows), whereas the RGC-32 KO mice showed scant EPHA7 co-localization in their astrocytes (**Figure 6B**, arrows). In addition, the total number of EPHA7/GFAP double-positive cells per microscopic field was significantly higher in WT mice than in RGC-32 KO mice (p<0.05; **Figure 6D**), suggesting that RGC-32 facilitates the expression of EPHA7 during the acute phase of EAE.

### RGC-32 Facilitates the Expression of Genes that Encode ECM-Related Proteins

In order to detect other potential genes that are differentially regulated by RGC-32 but were not observed in our previous analysis, we compared the TGF-β-regulated levels of the genes from our database in WT and RGC-32 KO astrocytes. This analysis revealed that the TGF-β-regulated levels of 29 genes were significantly higher in WT astrocytes than in RGC-32 KO astrocytes (log_2_ -fold change ≥ 0.6; FDR < 0.05) (**Supplementary File 5**). Pathway enrichment analysis of these genes revealed that they belong to cellular components related to ECM and microfibrils, and reactome pathway analysis revealed significant regulation of processes such as “ECM organization”, “elastic fiber formation”, and “degradation of the ECM” (**Supplementary Data**).

**Figure 7:**
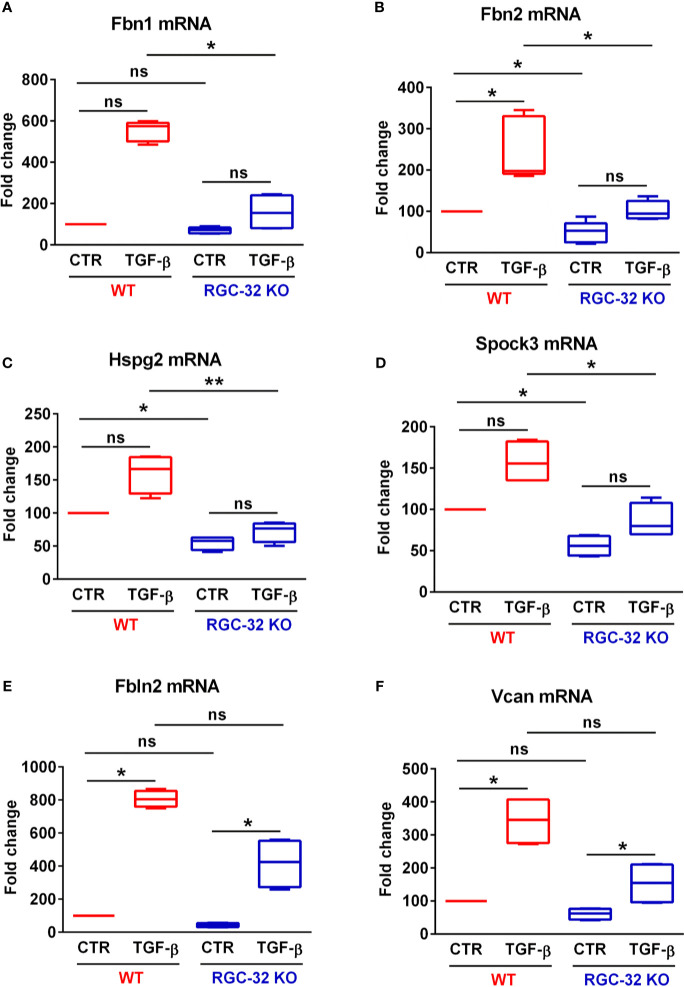
RGC-32 facilitates the *in vitro* expression of genes encoding ECM-associated fibrous proteins and proteoglycan core proteins. WT and RGC-32 KO mouse astrocytes were treated with 10 ng/ml TGF-β for 24 h. The expression of Fbn1, Fbn2, Hspg2, Spock3, Fbln2 and Vcan was then assessed by Real-Time PCR. Fbn1 **(A)**, Fbn2 **(B)**, Hspg2 **(C)** and Spock3 **(D)** were significantly higher in WT astrocytes than in RGC-32 KO astrocytes after TGF-β stimulation. There were also higher stimulated levels of Fbln2 **(E)** and Vcan **(F)** in WT astrocytes than in RGC-32 KO astrocytes, although these results did not reach statistical significance. The expression of the mRNA in unstimulated (CTR) WT astrocytes was considered to be 100, and the results are shown as -fold change. Data are expressed as mean ± SEM (N = 4). *p < 0.05; **p < 0.01; ns, not statistically significant (Kruskall-Wallis test with Dunn’s multiple comparisons test).

STRING network analysis of these 29 genes revealed that most of the functionally interconnected genes encode ECM-associated proteins belonging to families such as the fibrillins (Fbn1 and Fbn2), fibulins (Fbln2), and proteoglycan core proteins (versican [Vcan], heparan sulfate proteoglycan 2 [Hspg2]) (**Figure 4B**). Subsequent Real-Time PCR analysis confirmed the RNAseq findings, with the levels of Fbn1, Fbn2 and Hspg2 being significantly higher in WT astrocytes after TGF-β stimulation (**Figures 7A** **–C**). Another member of the proteoglycan core family, namely Sparc/osteonectin, cwcv and kazal-like domains proteoglycan 3 (Spock3), also known as testican-3, was also found to have higher levels in WT astrocytes than RGC-32 KO astrocytes (**Figure 7D**). On the other hand, we observed higher levels of Fbln2 and Vcan in WT astrocytes stimulated with TGF-β, although these results did not reach statistical significance (**Figures 7E** **, F**).

These findings suggest that RGC-32 is necessary to facilitate the transcription of a number of genes involved in ECM synthesis and deposition, with a particular emphasis on proteoglycan core proteins, a finding that further emphasizes RGC-32’s role in gliotic scar formation (17, 19, 38).

### RGC-32 Has an Inhibitory Effect on Genes Associated With Neural Stem Cells and Astrocyte Progenitors *In Vitro* and *In Vivo*

To unravel the most significant genes negatively regulated by RGC-32, we generated a list of genes whose TGF-β-regulated levels were significantly higher in RGC-32 KO astrocytes (**Supplementary File 6**) and a list of genes whose basal levels were significantly higher in RGC-32 KO astrocytes (**Supplementary File 7**). The two most significantly regulated genes in both categories were stanniocalcin 1 (Stc1), a gene up-regulated in gliomas (39) and WD repeat and FYVE domain-containing protein 1 (Wdfy1), a gene expressed in neural progenitors (40). Stc1 was also one of the 116 genes whose levels were significantly down-regulated by TGF-β in RGC-32 KO astrocytes (-fold change relative to unstimulated = 0.55; FDR = 0.03). Real-Time PCR results subsequently confirmed our RNAseq findings (**Figures 5G, H**) and showed that RGC-32 exerts an inhibitory effect on both Wdfy1 and Stc1 expression.

**Figure 8:**
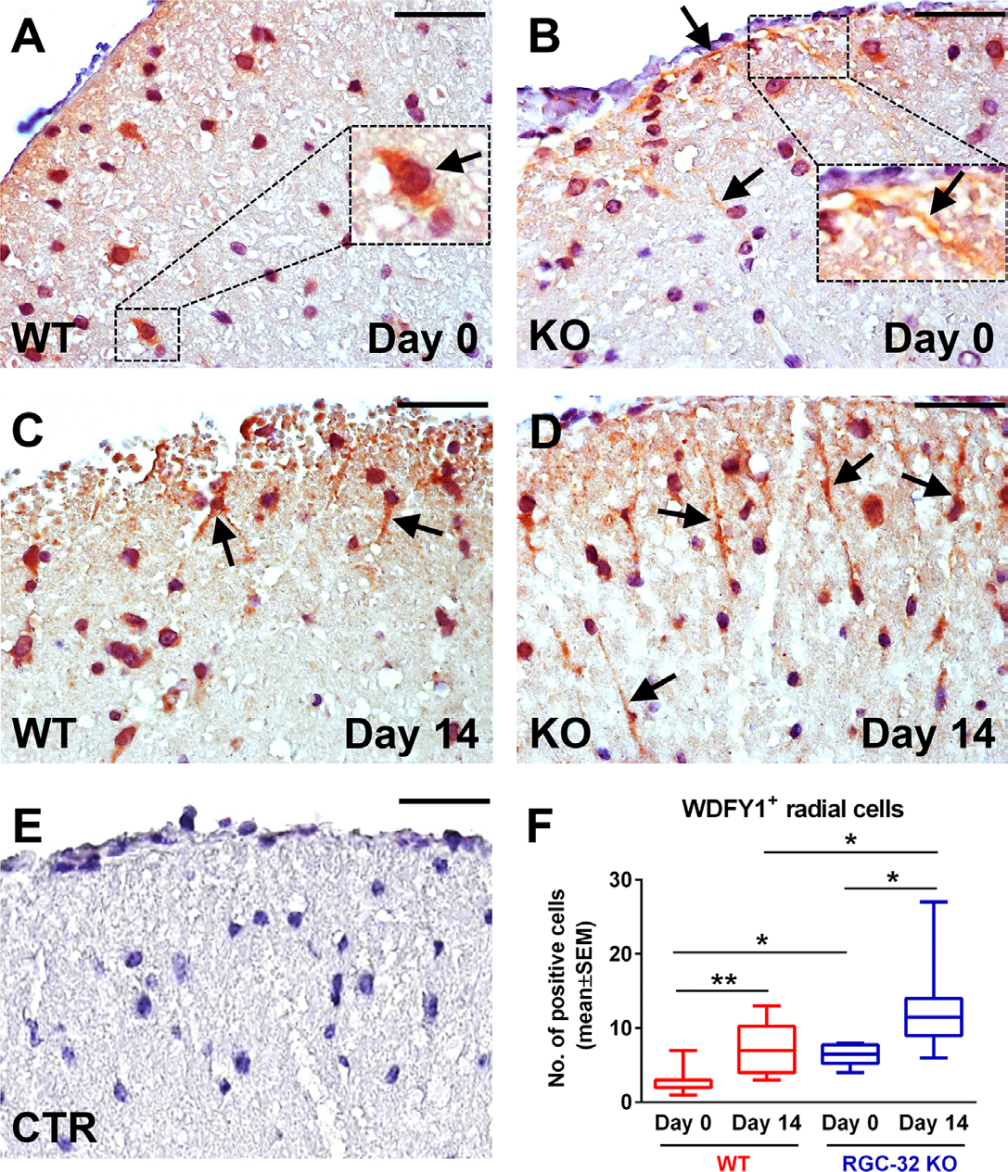
Lack of RGC-32 affects the number and morphology of WDFY1^+^ spinal cord cells *in vivo* in both normal adults and mice with EAE. Cervical spinal cords were harvested from control mice (day 0) and from mice with EAE on day 14, stained with an anti-WDFY1 antibody, and then counterstained with Harris hematoxylin. Day 0 WT mice were almost devoid of WDFY1^+^ cells with a bipolar shape, although some nuclear and perinuclear pattern was observed (**A**, arrow). RGC-32 KO mice (day 0) showed WDFY1^+^ radial glial cells; nevertheless, their processes were relatively short (**B**, arrows; number of WDFY1^+^ cells detected: 3). On the other hand, on day 14, WDFY1^+^ cells from RGC-32 KO mice had expanded their processes, which had become abundant (**D**, arrows; number of WDFY1^+^ cells detected: 9), in contrast to the WT mice, whose cell processes remained relatively short (**C**, arrows; number of WDFY1^+^ cells detected: 5). The control (CTR) for the immunoperoxidase reaction was negative **(E)**. Original magnification: x40. Scale bars: 20 µm. WDFY1^+^ cells with radial morphology were manually counted in white matter areas corresponding to x20-magnified spinal cord sections, and the results **(F)** are expressed as mean ± SEM. Day 0: n = 3 mice (8 microscopic areas) in each group. Day 14: N = 4 mice (10 microscopic areas) in WT; N = 4 mice (8 microscopic areas) in RGC-32 KO. *p < 0.05; **p < 0.01 (Kruskall-Wallis test with Dunn’s multiple comparisons test).

**Figure 9:**
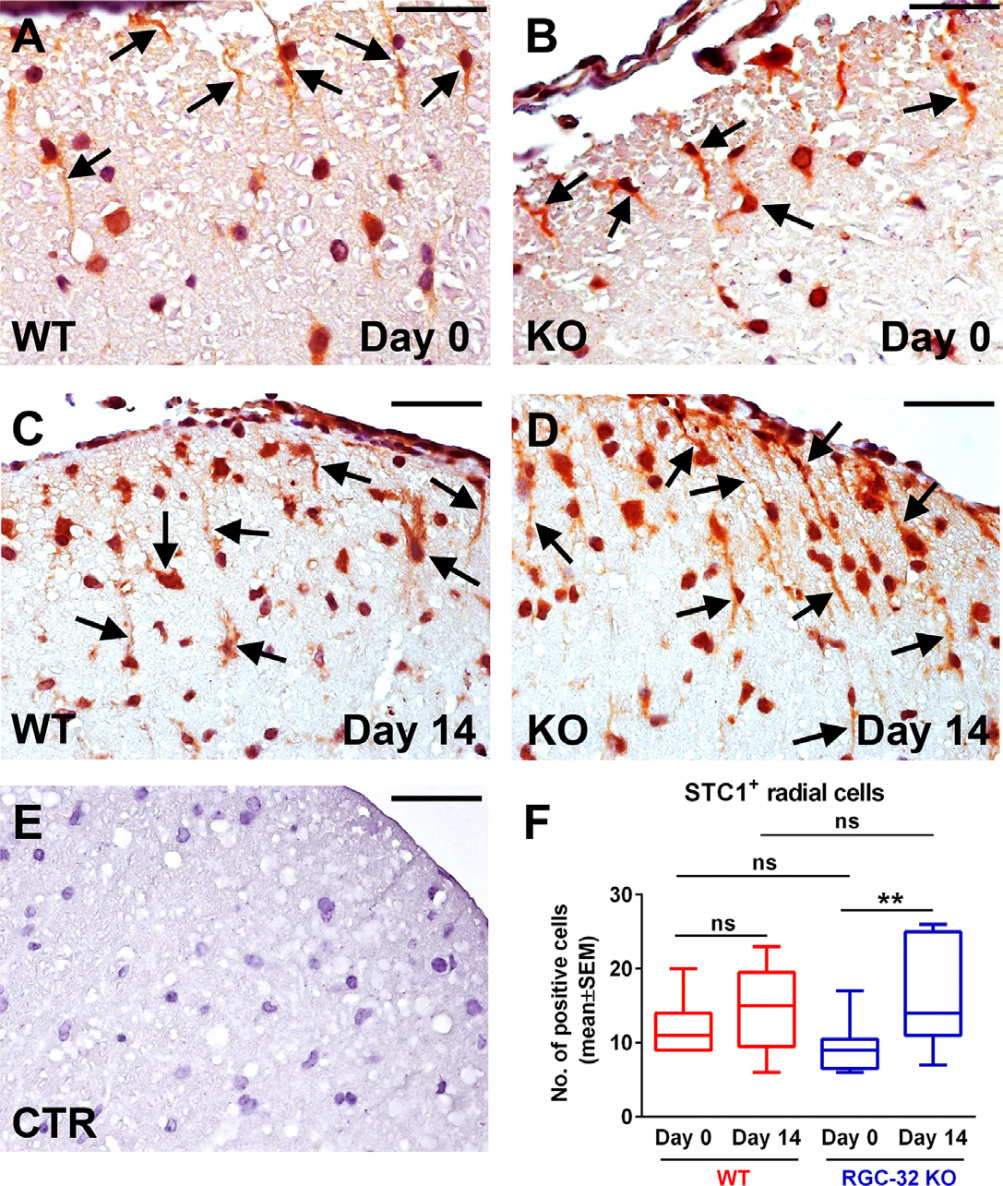
Lack of RGC-32 affects the number and morphology of STC1^+^ spinal cord cells *in vivo* at the peak of EAE. Cervical spinal cords were harvested from control mice (day 0) and from mice with EAE on day 14, stained with an anti-STC1 antibody, and then counterstained with Harris hematoxylin. We observed STC1^+^ radial glial cells in both WT (**A**, arrows; number of STC1^+^ cells detected: 8) and RGC-32 KO mice (**B**, arrows; number of STC1^+^ cells detected: 7) on day 0. On day 14, STC1^+^ cells from RGC-32 KO mice displayed an increase in the length of their processes (**D** arrows; number of STC1^+^ cells detected: 13), in contrast to WT mice, which showed little morphological change as compared to day 0 (**C** arrows; number of STC1^+^ cells detected: 9). The control (CTR) for the immunoperoxidase reaction was negative **(E)**. Original magnification: x40. Scale bars: 20 µm. STC1^+^ cells with radial morphology were manually counted in white matter areas corresponding to x20-magnified spinal cord sections. Only RGC-32 KO spinal cords showed a statistically significant increase in the number of these cells on day 14 as compared to day 0 **(F)**. The results in **(F)** are expressed as mean ± SEM. Day 0: n = 3 mice (9 microscopic areas) in each group. Day 14: N = 4 mice (13 microscopic areas) in WT; N = 4 mice (11 microscopic areas) in RGC-32 KO. **p < 0.01; ns, not statistically significant (Kruskall-Wallis test with Dunn’s multiple comparisons test).

These observations prompted us to further investigate WDFY1 and STC1 protein expression *in vivo* in normal mice and mice with EAE by using immunohistochemistry (**Figures 8A–F** and **9A–F**). On day 0, we observed a weak staining pattern for WDFY1 in the white matter of spinal cords, although the WDFY1^+^ cells with a radial morphology were more easily distinguishable in RGC-32 KO mice (**Figure 8B**, arrows). In comparison, WT mice were almost completely devoid of WDFY1^+^ cells with a bipolar shape, although some nuclear expression was observed (**Figure 8A**, arrow). On day 14, we noticed that the number of WDFY1^+^ radially shaped cells significantly increased in both types of mice as compared to day 0 (p<0.01 for WT; p<0.05 for RGC-32 KO; **Figure 8F**), and their processes became thicker and more elongated. Nevertheless, the cells from RGC-32 KO mice had even longer processes (**Figure 8D**, arrows) and were present in higher number (p<0.05; **Figure 8F**) than those in WT mice (**Figure 8C**, arrows).

When we analyzed the expression of STC1 on day 0, we observed STC1^+^ radial cells in both the WT and RGC-32 KO mice (**Figures 9A, B**, arrows). However, there was no significant difference in the number of these cells between the two groups (**Figure 9F**). Nevertheless, when we counted the total number of STC1^+^ cells on day 0, including those that showed only a nuclear or perinuclear pattern, we found that the RGC-32 KO mice had a slightly (albeit statistically significantly) higher number of these cells than did the WT mice [WT: median = 32.5, sum of ranks = 48; RGC-32 KO: median = 39.5, sum of ranks = 88; p = 0.03, Mann-Whitney test. N = 3 mice (8 microscopic areas) in each group]. On day 14, we observed that the STC1^+^ radial cells became more elongated in the RGC-32 KO mice than they were on day 0 (**Figure 9D**, arrows), whereas in WT mice these cells did not undergo any significant gross morphological changes between the two time points (**Figure 9C**, arrows). When we counted the number of STC1^+^ cells with a bipolar morphology, we found that there were more in RGC-32 KO mice on day 14 than on day 0 (p<0.01; **Figure 9F**), whereas in WT mice it remained roughly the same between the two time points.

Taken together, these results demonstrate that WDFY1^+^ and STC1^+^ radial glial cells increase in number in the spinal cords of mice that lack RGC-32 during acute EAE when compared to RGC-32 KO control mice.

### Lack of RGC-32 Affects the Number and Morphology of Adult Radial Glia at the Peak of EAE

**Figure 10:**
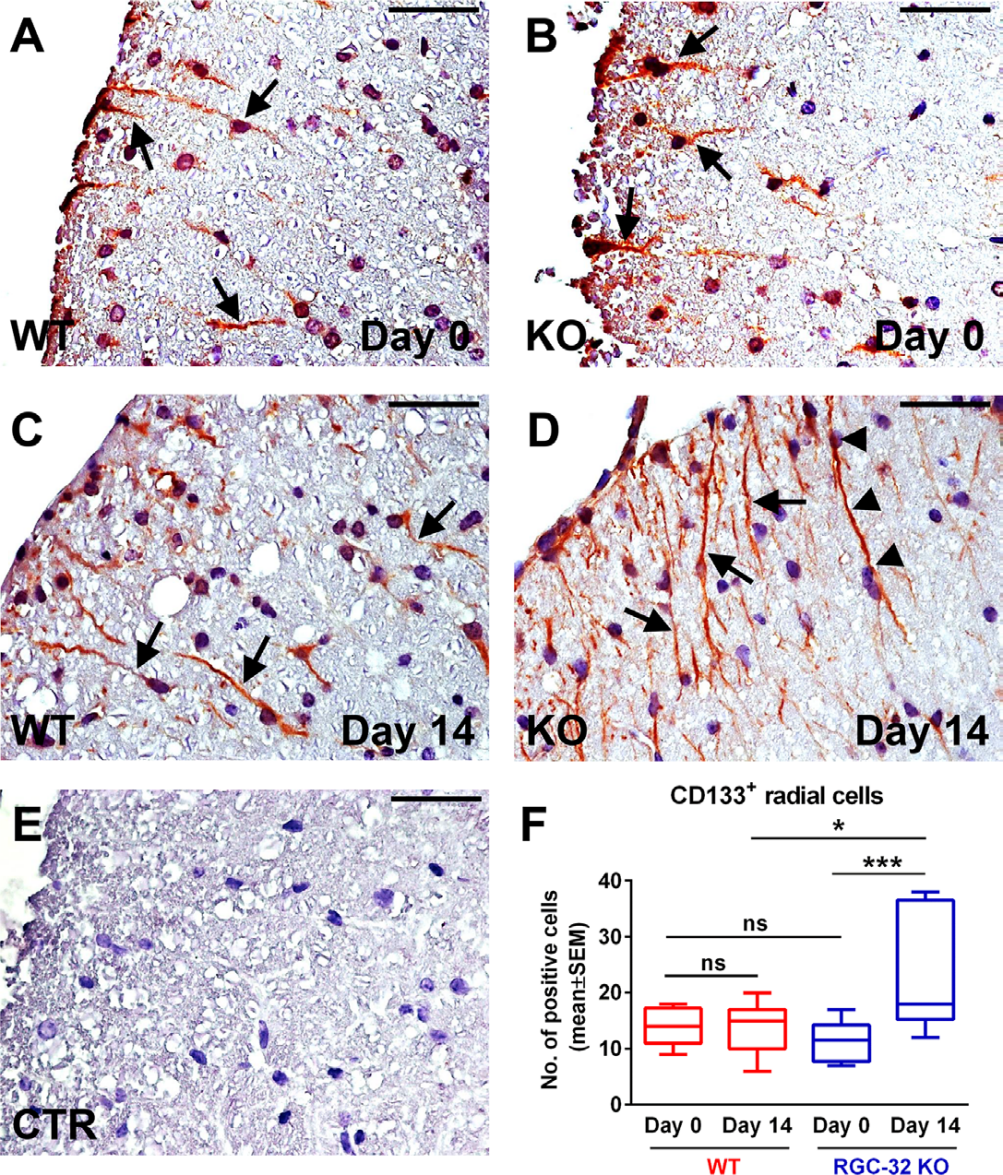
Lack of RGC-32 affects the number and morphology of CD133^+^ spinal cord radial glia *in vivo* during the acute phase of EAE. Cervical spinal cords were harvested from control mice (day 0) and from mice with EAE on day 14, stained with an anti-CD133 antibody, and then counterstained with Harris hematoxylin. On day 0, we did not see any noticeable difference in the morphology of the CD133^+^ cells with a radial shape between the WT and RGC-32 KO mice (**A, B**, arrows). On the other hand, on day 14, CD133^+^ cells from RGC-32 KO mice showed an expansion in the length of their processes (**D** arrows, some of them even showing a chain arrangement **(D)**, arrowheads), whereas in WT mice, these cells generally retained their morphology and length as compared to day 0 (**C**, arrows). The control (CTR) for the immunoperoxidase reaction was negative **(E)**. Original magnification: x40. Scale bars: 20 µm. Counting of the CD133^+^ cells with radial morphology revealed no difference between WT and RGC-32 KO on day 0, and a significantly higher number in RGC-32 KO mice than in WT mice on day 14. Also, only in RGC-32 mice did we detect a significant increase in the number of these cells on day 14 as compared to day 0 **(F)**. Results in F are expressed as mean ± SEM. Day 0: n = 3 mice (8 microscopic areas) in WT; n = 3 mice (10 microscopic areas) in RGC-32 KO. Day 14: N = 4 mice (11 microscopic areas) in WT; N = 3 mice (10 microscopic areas) in RGC-32 KO. *p < 0.05; ***p < 0.001; ns, not statistically significant (Kruskall-Wallis test with Dunn’s multiple comparisons test).

**Figure 11:**
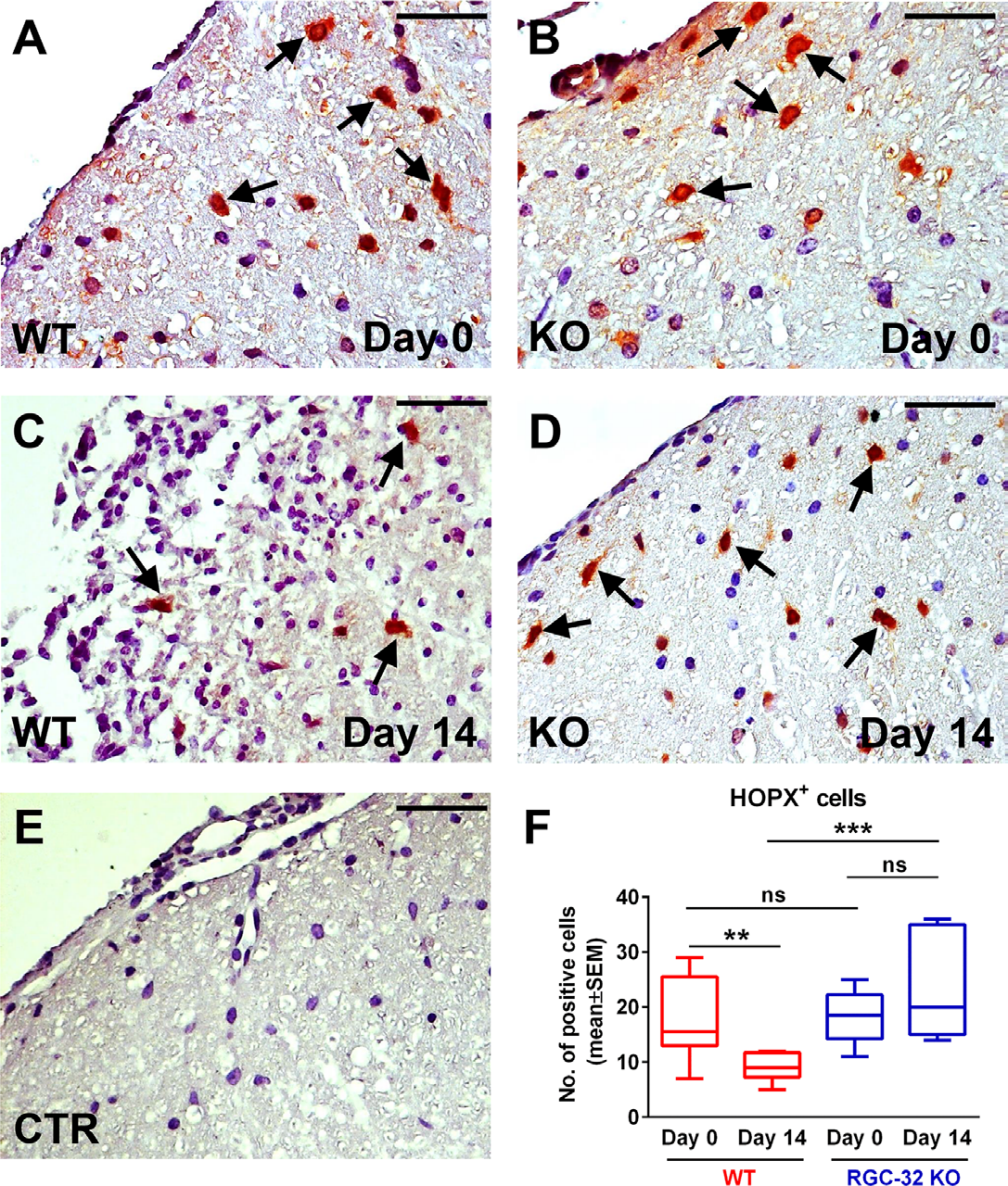
Lack of RGC-32 affects the number of HOPX^+^ spinal cord radial glia *in vivo* during the acute phase of EAE. Cervical spinal cords were harvested from uninjected mice (day 0) and from mice with EAE on day 14, stained with an anti-HOPX antibody, and then counterstained with Harris hematoxylin. On day 0, HOPX^+^ cells were distributed with a relatively equal density in WT and RGC-32 KO mice **(A, B)**, arrows. However, on day 14, the number of HOPX^+^ cells was significantly higher in RGC-32 KO mice **(D)**, arrows than in WT mice **(C)**, arrows. In WT mice, we detected fewer HOPX^+^ cells on day 14 than on day 0, whereas in RGC-32 KO mice we did not detect any noticeable difference between day 0 and day 14. The control (CTR) for the immunoperoxidase reaction was negative **(E)**. Original magnification: x40. Scale bars: 20 µm. Counting the total number of HOPX^+^ cells per microscopic field revealed no difference between WT and RGC-32 KO mice on day 0, and a significantly higher number in RGC-32 KO mice than in WT mice on day 14 **(F)**. Results in **(F)** are expressed as mean ± SEM. Day 0: N = 3 mice (8 microscopic areas) in each group. Day 14: N = 3 mice (8 microscopic areas) in each group. **p < 0.01; ***p < 0.001; ns, not statistically significant (Kruskall-Wallis test with Dunn’s multiple comparisons test).

To further investigate and better understand how RGC-32 regulates the development and dynamics of radial glia, we analyzed the distribution of two radial glial cell markers, CD133 and homeodomain-only protein homeobox (HOPX) (**Figures 10A–F** and **11A–F**). CD133 is a transmembrane cell adhesion protein that is present on a subpopulation of radial glia with pluripotent stem cell properties (41). CD133 immunostaining of spinal cords from uninjected mice (day 0) revealed the presence of CD133^+^ cells with short radial processes in the white matter of both WT and RGC-32 KO mice (**Figures 10A, B**, arrows). There was no significant difference in the number of these cells between WT and RGC-32 KO mice (**Figure 10F**). On the other hand, in the acute phase of EAE, RGC-32 KO mice showed an abundance of CD133^+^ radial glia when compared to the WT mice (p<0.05, **Figure 10F**), and the processes of these radial glia were more elongated than those of their WT counterparts (**Figures 10C**, 10, arrows). Some of these processes were arranged in chains perpendicular to the pial surface (**Figure 10D**, arrowheads). In contrast, we saw little to no morphological change in the CD133^+^ radial glia processes in WT mice between day 0 and day 14 (**Figures 10A, C**, arrows). Interestingly, we also detected a significant increase in the number of CD133^+^ radial glia in the RGC-32 KO mice at day 14 as compared to day 0 (p<0.001), whereas in the WT group, there was no change (**Figure 10F**).

We then analyzed the expression of HOPX, a small homeodomain protein expressed in adult radial glia destined to be astrogliogenic (42, 43). HOPX immunostaining revealed a mostly nuclear and perinuclear pattern. On day 0, we detected HOPX^+^ cells in both the WT and RGC-32 KO spinal cords (**Figures 11A, B**, arrows), with no significant numeric difference between the two groups (**Figure 11F**). However, on day 14, we observed a higher number of HOPX^+^ cells in the RGC-32 KO mice (**Figure 11D**, arrows) than in the WT mice (**Figure 11C**, arrows) (p<0.001, **Figure 11F**). Curiously, the number of HOPX^+^ cells was significantly decreased in the WT mice on day 14 as compared to day 0 (p<0.01), whereas in the RGC-32 KO mice, we detected a slight increase between day 0 and day 14, although this difference was not statistically significant (**Figure 11F**).

Collectively, these findings indicate that RGC-32 regulates the ability of radial glia and neural stem cells to undergo numeric and morphological changes during acute EAE and further strengthen RGC-32’s role in astrocytic development and the progenitor response to neuroinflammation.

## Discussions

Our RNA sequencing analysis has revealed that TGF-β stimulation of astrocytes turns on, at least partially, some of the transcriptional programs that are active during CNS development. The transcriptome analysis revealed for the first time that RGC-32 exerts an inhibitory effect on both STC1 and WDFY1. WDFY1 is an adaptor protein that has been found to potentiate the Toll-like receptor 3 and 4 (TLR3, TLR4) signaling pathways, thus contributing to innate immune responses (44). Although there is scarce evidence to suggest its involvement in brain processes, a couple of studies have found that WDFY1 is expressed in neurons and neural stem cells during differentiation and may contribute to neurogenesis (40). Yeo et al. have shown that peroxiredoxin inhibits neurogenesis by downregulating WDFY1 expression in neural precursor cells in a transgenic mouse model (40). Recently, Ouyang and coworkers have demonstrated that WDFY1 regulates dendritic morphogenesis in rodent pyramidal neurons (45). STC1 is a secreted glycoprotein that was initially described as a hormone involved in the regulation of calcium and phosphorus metabolism (46). Subsequent studies have shown that STC1 expression increases in gliomas and is associated with tumor development and progression (39, 46, 47). A study by Li et al. has demonstrated that overexpression of STC1 augments the stem-cell like properties of glioblastoma cells by increasing the NOTCH-SOX2 signaling pathway (48). Luo and coworkers have recently shown that STC1 levels directly correlate with those of matrix metalloproteinases (MMP)-2, MMP9, and vimentin in gliomas, and these authors hypothesized that STC1 augments the invasive capacities of glioma cells (49). We have also recently shown that RGC-32 KO astrocytes express higher levels of MMP2 and MMP9 than do WT astrocytes, possibly as a means of increasing ECM degradation and enhancing cell migration (19).

**Figure 12:**
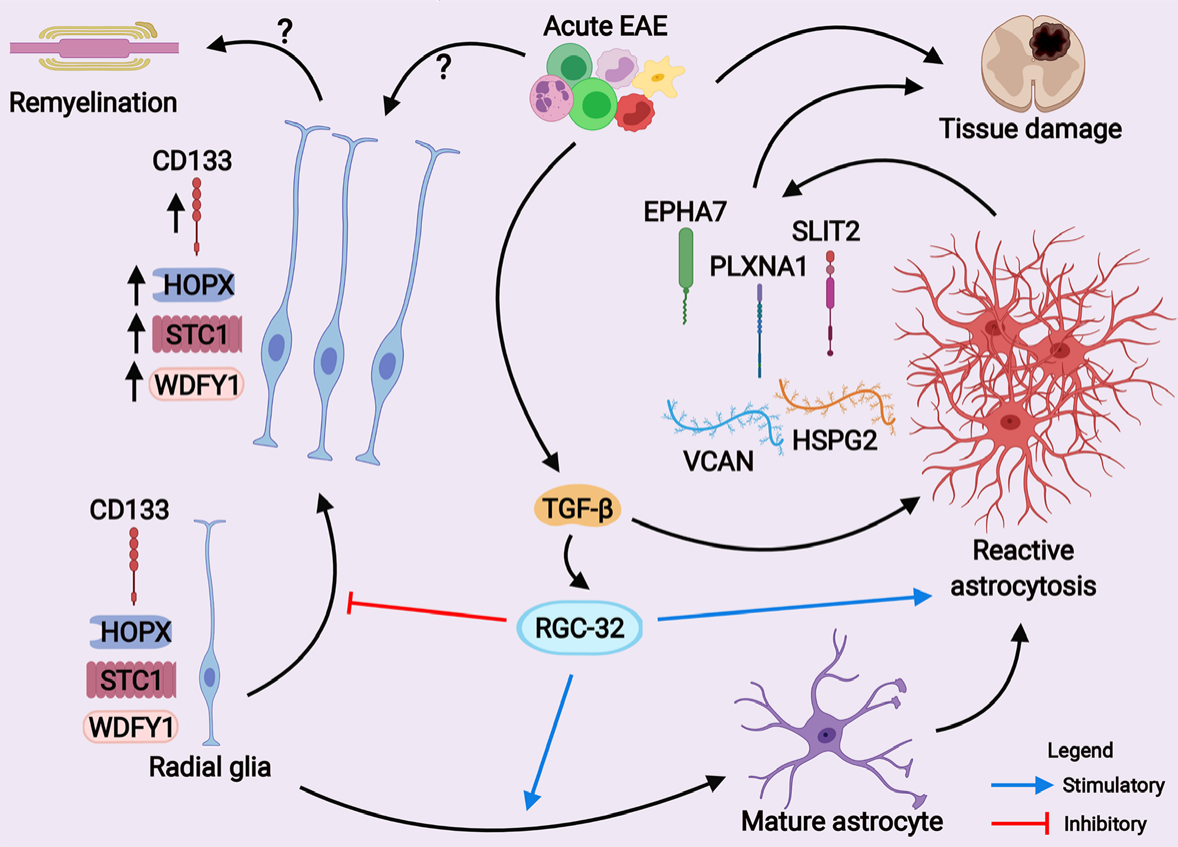
A scheme depicting the main hypothesis of how RGC-32 works as a regulator of astrocyte development and reactive astrocytosis during EAE. Our current results suggest that RGC-32 facilitates a pathogenic reactive phenotype of astrocytes during acute EAE. On the other hand, RGC-32 seems to negatively regulate the proliferation, morphological changes and possibly migration of CD133^+^, HOPX^+^, STC1^+^ and WDFY1^+^ radial glia at the peak of EAE, as these cells increase their number and become more elongated when RGC-32 is absent. However, whether these radial glia cells exert protective effects is yet to be established. (Created with BioRender.com).

To our knowledge, our study is the first to evaluate STC1 and WDFY1 expression in mouse astrocytes both *in vitro* and *in vivo* during EAE. The morphology and dynamics of WDFY1^+^ and STC1^+^ cells in the spinal cords of RGC-32 KO mice were highly similar to those of CD133^+^ radial glia, in that both types of cells increased their number and overall process length at day 14 of EAE as compared to day 0, and some of them even showed a chain arrangement (**Figure 10D**, arrowheads), a pattern similar to that described by others as a mean of migration for glial precursors from progenitor zones toward a lesion injury (43). CD133, also known as prominin-1, is a transmembrane glycoprotein expressed on the surface of neural stem cells in the developing and adult CNS and is a key histological marker for gliomas (50, 51). However, additional studies are necessary to establish whether STC1 and WDFY1 are co-expressed by these CD133^+^ radial glia (**Figure 12**). It is also important to mention that RGC-32 KO mice have an increased number of radial glia during acute EAE. This might be due to the fact that during acute EAE, other cytokines and growth factors regulated by RGC-32 might affect radial glia maturation and markers expression (19). On the other hand, in this study we did not find a difference in the number of CD133^+^ or HOPX^+^ radial glia (two subpopulations of radial glia) between WT and RGC-32 KO normal mice, suggesting that RGC-32 may differently regulate the expression of several markers of radial glia in normal *vs*. EAE mice.

Our study also revealed that a lack of RGC-32 significantly affects the number of HOPX^+^ cells in the spinal cords of mice with EAE in the acute phase. HOPX is an atypical transcription factor whose homeodomain lacks the ability to bind DNA; instead, it regulates DNA transcription indirectly through binding to other transcription factors (52). HOPX has been established as a marker of radial glia, and its expression is increased during neurodevelopment, as well as in the neurogenic niches of the adult brain such as the subventricular zone (42, 53, 54). A study by Vaid et al. has shown that HOPX is present in a subpopulation of neural progenitors called basal radial glia and is critical for maintaining their pool during neocortex development (55). Sweigel et al. identified HOPX as a key gene expressed by a subpopulation of neural stem cells that are mainly destined to differentiate into astrocytes in the adult subventricular zone (42). Another study has identified HOPX as one of the genes expressed in both neonatal and adult spinal cord radial glia (43). Interestingly, we observed that the number of HOPX^+^ cells was significantly decreased at the peak of EAE as compared to day 0 in WT spinal cords but was increased in RGC-32 KO spinal cords on day 14. We speculate that in WT spinal cords, most of the HOPX^+^ adult radial glia undergoes reactive changes in the acute phase of inflammation and loses HOPX expression, whereas RGC-32 KO radial glia increase their HOPX expression as a result of their inability to undergo reactive changes and adopt a mature phenotype, as we have recently demonstrated (19).

The up-regulation of AGM is of particular interest. The AGM comprise receptors, ligands, and cues that guide the growing axons toward their destination. These processes are critical for normal physiological processes such as axonal growth, neuronal migration, and synaptogenesis (56). However, some of these AGM are also up-regulated in response to CNS insults, and reactive astrocytes are regarded as a major source for their postnatal synthesis (57). Depending upon their ligand-receptor combinations, ACM can behave as either attractants or repellants for the inflammatory cells and exert both pro-inflammatory and anti-inflammatory properties (57).

Among AGM that are differentially regulated in WT astrocytes, we detected members of the ephrin receptor family, such as Epha7. Epha7 has been reported to be one of the several ephrin receptors that are up-regulated in reactive astrocytes from active MS lesions (37). Willson et al. have found that Epha7 has the highest immunoreactivity among ephrin receptors in the spinal cords of rats affected by thoracic contusion, and its expression is localized to glial cells (58). Figueroa et al. have also found increased Epha7 immunoreactivity in reactive astrocytes from injured spinal cords and observed that its inhibition accelerates functional recovery of the affected rats (59). In our study, we found that Epha7 was co-localized with astrocytes in WT mice at the peak of EAE, and this co-localization was decreased in RGC-32 KO mice. Based on these findings, we speculate that up-regulation of Epha7 is one of the molecular mechanisms through which RGC-32 facilitates reactive astrocytosis during EAE (**Figure 12**).

We have previously shown that in astrocytes, RGC-32 facilitates the nuclear translocation of STAT3, a transcription factor critical for the gliogenic switch during astrogliogenesis (19). In the present study, we observed that RGC-32 regulates the expression of genes that encode transcription factors (e.g., Runx2), cytokines (e.g., Il-11) and ECM proteins (e.g., Tenascin R) with potential astrogliogenic properties. For instance, a number of researchers have reported that the Runt-related transcription factor Runx2 is critical for astrocyte differentiation (60) and maturation (61). Another study has found that the IL-6 family member IL-11 is able to induce astrocytic differentiation in fetal neuroepithelial cells through activation of STAT3 (30). Tenascin R is a multifunctional glycoprotein composed of several domains that is involved in multiple processes in the CNS, such as neural stem cell migration, neuronal differentiation, synaptogenesis, cell adhesion, and neurite outgrowth (62). One of its regions containing fibronectin type III repeats has been shown to induce astrogliogenesis from neural stem cells, a process dependent on its interaction with β-1 integrin (63).

In conclusion, our findings suggest that RGC-32 might contribute to astrocytes maturation and reactivity during acute EAE, either directly or indirectly through its ability to regulate immune cell differentiation towards a pathogenic phenotype (18). Our data also show that RGC-32 is crucial for TGF-β’s ability to trigger transcriptomic changes in reactive astrocytes, which are then translated into complex molecular networks, ultimately leading to gliosis and tissue remodeling. Collectively, our data suggest that RGC-32 is a transcriptional hub that links molecular programs involved in astrogliogenesis and reactive astrocytosis, thus making it a strong candidate with therapeutic potential in MS.

## Data Availability Statement

The datasets presented in this study can be found in online repositories. The names of the repository/repositories and accession number(s) can be found below: https://www.ncbi.nlm.nih.gov/geo/, GSE173782.

## Ethics Statement

The animal study was reviewed and approved by University of Maryland School of Medicine Office of Animal Welfare Assurance.

## Author Contributions

AT, VR, TB, and HR designed the study. AT, AB, VN, DB, CC, and TB performed the experiments. AT, DM, VR, TB, and HR wrote the manuscript. All authors contributed to the article and approved the submitted version.

## Funding

This work was supported in part by a grant from Veterans Administration Merit Award (I01BX001458 to HR) and by an RO1 NS42011 grant (to HR). Austin Beltrand was supported in part by a medical student research grant from the Foundation of the Consortium of Multiple Sclerosis Centers’ MS Workforce of the Future and by the Proposed Research Initiated by Students and Mentors (PRISM) program, University of Maryland School of Medicine Office of Student Research, Baltimore, USA.

## Conflict of Interest

The authors declare that the research was conducted in the absence of any commercial or financial relationships that could be construed as a potential conflict of interest.

## Publisher’s Note

All claims expressed in this article are solely those of the authors and do not necessarily represent those of their affiliated organizations, or those of the publisher, the editors and the reviewers. Any product that may be evaluated in this article, or claim that may be made by its manufacturer, is not guaranteed or endorsed by the publisher.

## Abbreviations

AGM, axonal guidance molecules; BBB, blood-brain barrier; BP, biological process; CNS, central nervous system; DEG, differentially expressed genes; EAE, experimental autoimmune encephalomyelitis; ECM, extracellular matrix; EGFL6, epidermal growth factor-like domain-containing protein 6; EPHA7, ephrin receptor type 7A; Fbn, fibrillin; Fbln2, fibulin 2; FDR, false discovery rate; GFAP, glial fibrillary acidic protein; Hspg2, heparan sulfate proteoglycan 2; HOPX, homeodomain-only protein homeobox; Itgb1, integrin beta 1; KO, knock-out; MMP, matrix metalloproteinase; MS, multiple sclerosis; Plxna1, plexin A1; RGC-32, response gene to complement 32; Spock3, Sparc/osteonectin, cwcv and kazal-like domains proteoglycan 3; STAT3, signal transducer and activator of transcription 3; STC1, stanniocalcin-1; Tnr, tenascin R; Vcan, versican; WDFY1, WD repeat and FYVE domain-containing protein 1; WT, wild type.
