# Supplementary material for: RGC-32 Acts as a Hub to Regulate the Transcriptomic Changes Associated With Astrocyte Development and Reactive Astrocytosis
Source: Front Immunol. 2021 Jul 29;12:705308. doi: 10.3389/fimmu.2021.705308 (PMC8358671; doi:10.3389/fimmu.2021.705308)
Supplement: Supplementary file 1 [file DataSheet_1.zip › Supplementary table + figure.docx]

| **Supplementary table 1 \| Mouse primers used for Real Time PCR** | | |
| --- | --- | --- |
| **Gene symbol** | **Primers sequence** | |
| Egfl6 | For: 5’- GCAAATGCAAGCAGGGATATAG -3’ | |
|  | Rev: 5’- CCCAGGTGCTGTGAGTATTT -3’ | |
| Epha7 | For: 5’- GGATGAGAACTACACTCCGATAAG -3’ | |
|  | Rev: 5’- CCTTTGTGCGTTGCCTTTAG -3’ | |
| Fbln2 | For: 5’- CAGTCCTGTGAGTCCAACCC -3’ | |
|  | Rev: 5’- CTCTCGTGCAGTGTCCAACT -3’ | |
| Fbn1 | For: 5’- CAGGCTCTTCTGTGTCGATATT -3’ | |
|  | Rev: 5’- TGGCTGACAGCTACATTCATAG -3’ | |
| Fbn2 | For: 5’- GAGCACAATGAGGACGACTAC -3’ | |
|  | Rev: 5’- CAAGTCTGCAGAGGGCTAATAC -3’ | |
| Hspg2 | For: 5’- ATCTGTAACGCCACCAACTC -3’ | |
|  | Rev: 5’- GCCGAGGTATGTTCTGGAATTA -3’ | |
| Itgb1 | | For: 5’- GACAGTGTGTGTGTAGGAAGAG -3’  Rev: 5’- GCCTCCACAAATTAAGCCATTAG -3’ |
| Plxna1 | | For: 5’- TGTACTGAAGGAGATGGAGGT -3’  Rev: 5’- CTAGTGTGCGGATGAAGGTTAG -3’ |
| Runx2 | | For: 5’- TGGCTTGGGTTTCAGGTTAG -3’  Rev: 5’- GGTTTCTTAGGGTCTTGGAGTG -3’ |
| Slit2 | | For: 5’- CTGTGCGTCTGCTCTCTTTAT -3’  Rev: 5’- AGGATTGGCCAAGAGGTTTAG -3’ |
| Spock3 | | For: 5’- GGCCTGAGAGAAGCAGATTT -3’  Rev: 5’- CTGGTCCAATAGCAGGTCATAG -3’ |
| Stc1 | | For: 5’- CCTATCTACTCTCCGCAAACAC -3’  Rev: 5’- CTAACTCTACCAGGTGACCATTC -3’ |
| Vcan | | For: 5’- AGCTACGGAGCTCTTCAGGA -3’  Rev: 5’- AGATCACATAGGAAGCGCGG -3’ |
| Wdfy1 | | For: 5’- GGATATTTGTGGGCCAGGATAA -3’  Rev: 5’- TGGGCTGGATAGGTCTTGATA -3’ |
| 18S | For: 5’- GTAACCCGTTGAACCCCATT -3’ | |
|  | Rev: 5’- CCATCCAATCGGTAGTAGCG -3’ | |

***Supplementary Material***

**1. Tables**

**2. Figures**

**
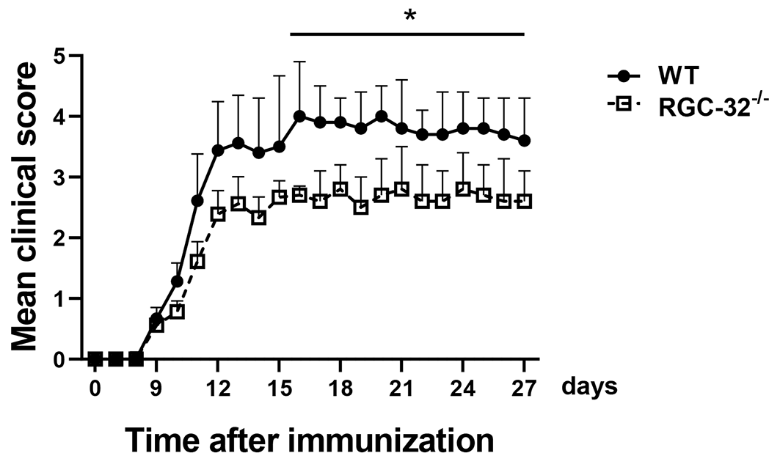
**

**Supplementary Figure 1. Lack of RGC-32 attenuates the clinical course of EAE**

WT and RGC-32 KO female mice were immunized with MOG_35–55_ and then scored as described in Materials and Methods. Data are expressed as mean ± SEM and are representative of three independent experiments (6 mice for each group/ experiment). * = p<0.05.
